# Infection of prepubertal heifer calves as a natural host model for *Tritrichomonas foetus*

**DOI:** 10.3389/fcimb.2025.1628192

**Published:** 2025-10-15

**Authors:** Katy A. Martin, Sara Reece, Jeba R. J. Jesudoss Chelladurai, Tyler A. Harm, Jodi D. Smith, Courtney N. Blake, Douglas E. Jones, Kris Kovach, Krysta McMahan, Erica Moscoso, Morgan Ostrander, Matthew T. Brewer

**Affiliations:** ^1^ Department of Veterinary Pathology, Iowa State University College of Veterinary Medicine, Ames, IA, United States; ^2^ Department of Clinical Sciences, Auburn University College of Veterinary Medicine, Auburn, AL, United States; ^3^ National Veterinary Services Laboratory, Animal and Plant Health Inspection Service, United States Department of Agriculture, Ames, IA, United States; ^4^ Practical Livestock Services, Casey, IA, United States

**Keywords:** sexually transmitted infections, parasitology, animal models, trichomonosis, bovine reproductive diseases

## Abstract

**Introduction:**

*Tritrichomonas foetus* is a sexually transmitted flagellate that causes economic loss in the cattle industry throughout the world. In the United States, there are no approved treatments for the parasite. Owing to its transmission strategy, *T. foetus* typically infects cattle of breeding age. However, *in vivo* studies of treatment, diagnostic strategies, and vaccination are severely hampered by the maintenance and cost of maintaining adult cattle in research settings. In this study, we investigated the utility of infecting pre-pubescent heifer calves with *T. foetus*.

**Methods:**

Four independent cohorts of cross-bred prepubertal heifer calves were vaginally inoculated with *T. foetus* trophozoites previously derived from a naturally-infected bull. Infections were assessed by culture, PCR, DNA sequencing, histopathology, gross pathology, and lesion scoring. In addition, reproductive tract tissue was assessed for the presence of galectin-1, a putative receptor for *T. foetus* trophozoite adhesion.

**Results:**

Our experiments revealed that despite being in anestrus, heifer calves were amenable to infection with trophozoites for as long as 42 days post-infection as determined by PCR and culture of the organism. Histopathology revealed inflammation throughout the reproductive tract of infected calves. Infection resulted in endometritis with lymphoplasmacytic infiltration and demonstrated that trophozoites could pass through the cervix even during anestrus in prepubescent heifers. In addition, immunohistochemistry of the vagina, cervix, and uterus demonstrated robust expression of galectin-1.

**Conclusion:**

Our experiments demonstrated that prepubertal heifer calves are a suitable natural host model for bovine trichomonosis. This is a significant breakthrough in the field and also has potential for advancing the human trichomoniasis research agenda.

## 1 Introduction

*Tritrichomonas foetus* is a sexually transmitted protozoan parasite of cattle. Bulls typically serve as chronic asymptomatic carriers, while naïve heifers and cows are infected during breeding. Infection of female cattle results in inflammation throughout the reproductive tract, early embryonic death, and less commonly, abortions (BonDurant, 1997). Ultimately these lost pregnancies lead to a widespread calving interval when cows clear the infections and go on to conceive in subsequent estrus cycles (Ortega-Mora et al., 2022). The impact of bovine trichomonosis is primarily economic; widespread calving intervals result in non-uniform calf crops which are less valuable for cattle operations (Rae, 1989; Yao, 2021). The use of artificial insemination practically eliminates the risk of transmission and infection with *T. foetus* (Bernasconi et al., 2014). While artificial insemination is used almost exclusively among dairy operations, it is impractical for many beef cattle producers who rely on bulls for estrus detection and breeding of cows (Fernandez-Novo et al., 2020). There are no approved treatments for bovine trichomonosis in the US (Martin et al., 2020; Martin et al., 2023). A killed vaccine is available but fails to prevent infection (Baltzell et al., 2013). Control efforts rely on testing and culling infected animals while a patchwork of regulations prevents movement of infected cattle across state lines (Martin et al., 2021). In order to develop and approve efficacious treatments or vaccines for bovine trichomonosis, animal studies in research settings are required.

Research progress regarding *T. foetus* in the bovine host is hampered by the challenge of housing mature cattle in research settings. Experimental *T. foetus* infections in cows and heifers have previously relied on hormone synchronization to mimic natural infections, which occur during estrus when females are receptive to breeding by bulls. Cattle typically undergo puberty by 12 months of age (Rawlings et al., 2008; Day and Nogueira, 2013). By this age, cattle are too large to be safely contained in many research facilities. Although estrus and ovulation occur prior to infection under natural settings, it is not known if these hormonal influences are actually a requirement for colonization of the female bovine reproductive tract with *T. foetus* trophozoites. In this study, we challenge the dogma that infection requires reproductive tract changes associated with the hormonal influence of estrus such as high estrogen and low progesterone (Sugarman and Mummaw, 1988; Bondurant, 1999; Lehker and Sweeney, 1999; Rutkowski and Harmsen, 2007).

While adhesion to the host is essential for the parasite to obtain nutrients, the molecular ligands that *T. foetus* trophozoites use to attach to bovine host cells have remained elusive. Parasite lipophosphoglycan has been partially implicated in the process of parasite attachment, as 2 μg of purified LPG inhibits parasite binding to bovine vaginal epithelial cells by 40 – 50% (Singh et al., 1999). However, specific chemical residues responsible for binding have not been identified. In addition, sialic acid residues have been identified on the surface of *T. foetus* and were thought to play a role in adhesion (Babál and Russell, 1999). However, neuraminidase treatment of trophozoites did not impair adhesion to MDCK cells, reducing support for the hypothesis that *T. foetus*-host cell binding is mediated by sialic acid (Silva-Filho and de Souza, 1988). Cysteine proteases have also been implicated in the host cell adhesion process of trichomonads. It is hypothesized that cleavage of parasite associated adhesins or other factors by proteases early in infection is a necessary step to promote parasite adhesion to host cells (Arroyo and Alderete, 1989). *Trichomonas vaginalis*, a related parasite and the cause of trichomoniasis in humans, is known to bind galectin-1 on host cells, with adherence decreasing by 20% when binding is inhibited (Okumura et al., 2008). Galectin-1 is encoded in the bovine genome and known to be expressed in a variety of tissues including lung, intestines, and spleen, with expression profiles varying with host age (Ahmed et al., 1996; Kaltner et al., 2002). To our knowledge, the expression of galectin-1 in the mucosa of the reproductive tract of prepubescent animals has not been previously investigated. In this study we also investigated the presence of galectin-1 in the reproductive tract of pre-pubescent heifers to determine if this molecule may be involved in *T. foetus* host cell adhesion.

Although *T. foetus* is a significant reproductive disease, the research agenda aimed to address this pathogen is hampered by the fact that housing mature cattle in research settings is challenging and simply not possible for many facilities. A more manageable infection model utilizing younger cattle would create significant research opportunities. In this study, we test the hypothesis that prepubescent calves can serve as a suitable natural host model for bovine trichomonosis. We aimed to determine if this age group of heifers is amenable to colonization by *T. foetus* trophozoites, resolve which regions of the reproductive tract express galectin-1, and assess the microscopic lesions associated with infection.

## 2 Materials and methods

### 2.1 Parasites

*Tritrichomonas foetus* trophozoites were maintained in trypticase-yeast extract-maltose (TYM) medium supplemented with adult bovine serum (10%) and 100X penicillin-streptomycin (1%) (Martin et al., 2021). Cultures were maintained at 35°C and regularly subcultured to maintain cell concentrations of approximately 4x10^5^ trophozoites/mL (Schaut et al., 2017). Cultures were maintained in sterile 15 mL centrifuge tubes filled completely with media and capped tightly to create an anaerobic environment. All experiments utilized IA-1 (field) strain parasite (Bader et al., 2016). Studies were conducted under all applicable local laws and approved by the Iowa State University Biosafety and IACUC committees under protocols 18–217 and 21-264.

### 2.2 Animals

Cross bred Holstein-Angus heifer calves (70 kg, 6–8 weeks of age) were purchased from commercial dairy operations in Iowa, USA. No twins or twin sibling calves were used in the study. Four independent experiments (A – D) were conducted, each at a different point in time. In experiments A and B, calves were group housed indoors in a controlled ambient environment. In experiments C and D, calves were group house in an outdoor environment. Experiment A included 3 heifer calves, experiments B and C included 6 heifer calves, and experiment D included 12 heifer calves. Experiment A utilized a minimal number of animals to form a basis for power analyses to determine the number of animals needed in subsequent studies. In subsequent experiments the proportion of animals infected and parasite counts were used to determine the number of animals required.

### 2.3 Experimental infections

**Figure 1:**
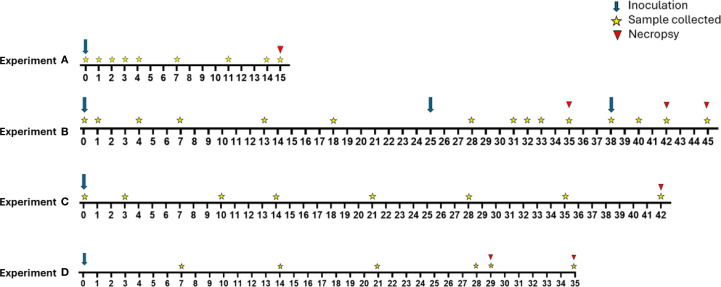
Overview of timelines for experimental groups A-D. Experiment A (n=3) included a single inoculation on day 0. Calves were euthanized and necropsied on day 15 post inoculation. Experiment B (n=6) included initial inoculation of all calves on day 0 with calf B.D being reinoculated on day 25 and calves B.A and B.F being reinoculated on day 38. Calves were euthanized on days 35 (B.C and B.E), 42 (B.B and B.D), and 45 (B.A and B.F). Experiment C (n=6) included a single inoculation on day 0. Calves were euthanized and necropsied on day 42. Experiment D (n=12) included a single inoculation on day 0. Calves were euthanized on days 29 (D.A, D.G, and D.H) and 35 (remaining calves).

An overview of the procedural timeline for each experiment is shown in **Figure 1**. Using a 15-gauge feeding needle attached to a syringe, heifers were infected with trophozoites (experiment A = 1x10^8^, B = 4.3x10^7^, C = 9.4x10^8^, D = 2x10^8^) in a total volume of 1.5–2 mL TYM delivered directly into the vaginal vault. Variation in the infectious dose was due to availability of parasite cultures at the time of each experiment. In experiment B calves with consecutive negative cultures were reinfected with 3.75x10^7^ (calf B.D) or 3.1x10^8^ (calves B.B and B.D) trophozoites. The rationale for reinfection was that it would not be worthwhile to sacrifice a negative animal for necropsy; such animals would not allow us to determine which parts of the reproductive tract were colonized.

To assess infection status, calves were sampled by swabbing the vaginal vault with a TYM-soaked cotton tip applicator (15cm) inserted and retracted 4 times while simultaneously rotating the applicator. The cotton tip applicator was placed into a microcentrifuge tube containing 1.5 mL TYM culture medium supplemented with Modified Preston Campylobacter selective supplement (Oxoid LTD, Hampshire, England).

### 2.4 Parasite culture

**Figure 2:**
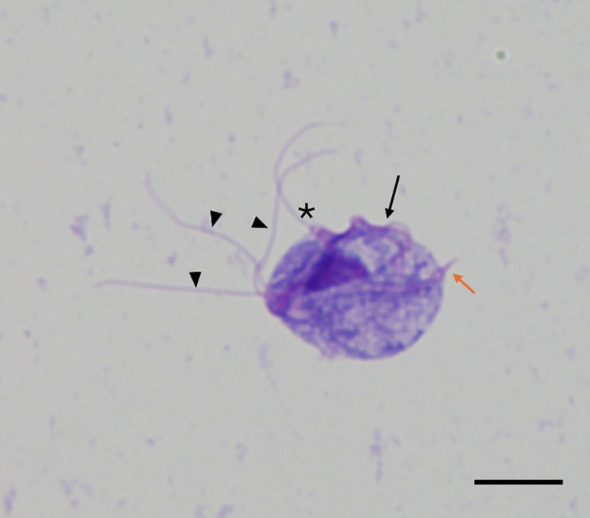
Wright-Giemsa stain of IA-1 *Tritrichomonas foetus* trophozoite recovered from growth media. Trophozoites were identified morphologically in culture samples. Trophozoites were approximately 10–25 µm in length with three anterior flagella (arrowheads), one posterior flagellum (asterisk), an undulating membrane (arrow), and an axostyle (orange arrow). (Magnification= 1000x, scale bar=10 μm).

Reproductive tract swabs were placed in TYM culture media, maintained at 35°C and observed microscopically every 24 hrs until motile *T. foetus* trophozoites were observed. Cultures were considered negative if no live organisms were observed within 7 days. Live organisms were identified morphologically. **Figure 2** provides an example of a typical trophozoite as observed in this study. Viable organisms demonstrated forward motion. Positive cultures were verified as needed by PCR described below.

### 2.5 PCR

Aliquots of all samples from experiment A vaginal swabs were submitted to the Iowa State University Veterinary Diagnostic Laboratory for PCR testing. In subsequent studies (B and D), culture positive samples were subjected to confirmatory PCR. DNA was extracted from 500 µL aliquots of culture media inoculated with sample swab using the QIAmp DNA Mini kit (Qiagen), in accordance with manufacturer instruction for cells in suspension. Conventional PCR using published primer pair TFR3/TFR4 was performed (**Table 1**) (Felleisen et al., 1998). Negative culture samples were used as a negative control in these reactions. PCR was carried out in a 20 μL volume with 2 μl DNA template, 0.5 µM forward and reverse primers, and 1x Platinum™ SuperFi™ II PCR Mastermix (Invitrogen). PCR conditions consisted of initial denaturation at 98°C for 30s, followed by 30 cycles consisting of 10s denaturation (98°C), 10s annealing (60°C), 1 min elongation (72°C); final elongation was carried out for 5 min (72°C). Agarose gel electrophoresis was performed to confirm amplification of bands of the expected size (~350 base pairs). Culture material from experiment C was utilized for transcriptomic analysis used in a separate study; PCR was not performed on samples from this experiment.

**Table 1:** PCR primer sequences.

Primer pair	Sequences	Reference
TFR3/TFR4	F: 5’-CGGGTCTTCCTATATGAGACAGAACC-3’ R: 5’- CCTGCCGTTGGATCAGTTTCGTTAA-3’	Felleisen et al. (1998)
S0295/S0296	F: 5’-CGAAAGGTCACGGATACACA-3’ R: 5’-CCCCATGAGTTTCTCACGAT-3’	Šlapeta et al. (2012)

In experiment A, DNA was also extracted from fresh vaginal, cervical, and uterine tissue samples collected at necropsy using the Qiagen DNeasy Blood and Tissue kit (Valencia, CA) according to manufacturer’s instructions for tissue samples. An approximately 700-bp fragment of T*. foetus* cysteine protease 2 (TfCP2) was amplified using the published primer pair S0295/S0296 (**Table 1**) (Šlapeta et al., 2012). PCR was carried out in a 25 μL volume with 2 μl DNA template, 1x PCR buffer (GoTaq Flexi buffer, Promega, Madison WI), 3 mM MgCl2, 0.2 mM dNTPs, 0.5 μM of each primer, and 2 U of Taq polymerase (GoTaq Flexi, Promega, Madison WI). PCR conditions were as follows: initial denaturation at 95°C for 5 min followed by 35 cycles of 15s denaturation (95°C), 15s annealing (55°C), and 30s elongation (72°C), with a final elongation step of 5 min at 72°C. Agarose gel electrophoresis was performed to visualize amplicons.

### 2.6 Histopathology

Samples of vaginal vault, cervix, and uterus were collected from calves at necropsy. Samples were fixed in 10% neutral buffered formalin (NBF), embedded in paraffin blocks, and processed through routine histologic methods (Chelladurai et al., 2021). Samples were H&E stained with Signature Series™ hematoxylin (Fisher Scientific) for five min and Richard-Allan Scientific™ eosin (Fisher Scientific) for one minute. Slides from group D were evaluated by an ACVP boarded pathologist (TAH). Group D was selected for histopathologic review because these animals were subject to fewer sampling instances, reducing the possibility of iatrogenic inflammation. Gram and Grocott’s methenamine silver (GMS) staining were performed on all tissues to rule out bacterial and fungal causes of inflammation.

### 2.7 Galectin-1 immunohistochemistry

Immunohistochemistry was performed to investigate the presence of galectin-1 on vaginal, cervical, and uterine tissue samples. Bovine trachea and lung, known to express galectin-1, were used as positive controls (Kaltner et al., 2002). For all IHC procedures, five-micrometer sections on positively charged slides were utilized. Antigen retrieval was performed using Decloaker (Biocare Medical, DV2004) in a decloaking chamber. Tissues were blocked with Peroxidase Blocking Reagent (Biocare Medical, IPB5000G20) for 5 min and Background Punisher (Biocare Medical, IP974G20) for 5 min. Galectin-1 Recombinant Monoclonal antibody, JM13-37 (Invitrogen, catalog number MA5-32779) was applied at a dilution of 1:400 in DaVinci Green (Biocare Medical, PD900H) with a 2-hour incubation. Rabbit-on-Canine HRP Polymer (Biocare Medical, RC542L) was used as a secondary with a 30-minute incubation. Signal was visualized using DAB Chromagen Kit (Biocare Medical, IPK5010) incubated on tissues for 5 min. Tissues were counterstained with a 1:10 dilution of CAT Hematoxylin (Biocare Medical, CATHE-M) for 5 min, dehydrated in a series of graded ethanol, and coverslipped.

## 3 Results

### 3.1 Infection persists in experimentally infected heifer calves for at least 42 days

**Figure 3:**
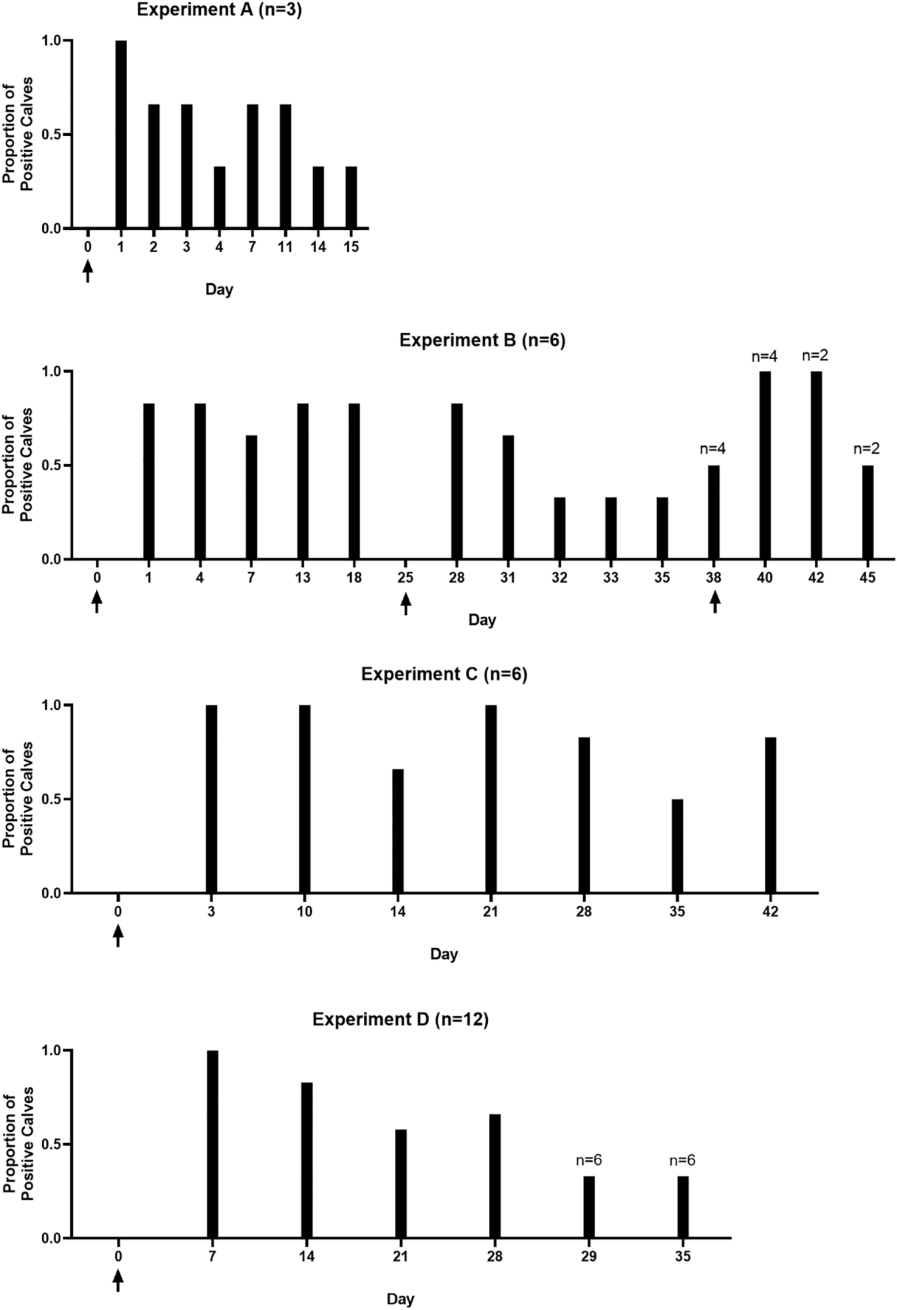
Calves remain culture positive for up to 42 days post-infection. Graphs demonstrate the proportion of calves in experiments A – D that were culture positive at each sampling timepoint. Group A included 3 calves. Groups B and C included 6 calves. Group D included 12 calves. Arrows indicate inoculation timepoints. Calves were not sampled on day 25 in experiment B, this timepoint is included in the graph as calf B.D was reinoculated on that day. Number of calves are given above the graph for each individual experiment; numbers above bars indicate the number of calves on days where less than the full experimental group was sampled.

Heifer calves remained infected for as long as 6 weeks (42 days), following a single inoculation (**Figure 3**). Since calves were sacrificed at this point, the total possible duration of infection in juvenile heifers is unknown. Results from the present series of experiments show that at least 60% of prepubescent heifer calves are culture positive at 4 weeks post inoculation, when experiments were carried out through this timepoint. Additionally, in the longer-term, single inoculation experiment (C), 5/6 calves were still culture positive at the 6-weeks post infection. Because samples were incubated for up to 7 days to determine infection status, enrichment time varied among samples, therefore, comparing the proportion of culture positive animals is more meaningful than the number of trophozoites recovered (**Figure 3**). The duration of experiments in this study varied. The rationale was that after we confirmed we could infect heifers with the parasite, we wished to verify specific parts of the reproductive tract that were colonized. As the duration of the infection became apparent, subsequent experiments were allowed to proceed for a longer timeframe. The rationale for not observing the time needed to clear the infection as is seen in naturally infected animals is that the location of parasites in the reproductive tract could not be determined if the host eliminated the infection.

### 3.2 *Tritrichomonas foetus* is present throughout reproductive tract following experimental infection

Vaginal, uterine, and cervical tissue samples collected from the calves in Experiment A were PCR positive for *T. foetus* using TfCP2 primers (**Table 2**). In experiment C, cultures collected from the lumen of the uterus of 3/6 calves at necropsy demonstrated live organisms, confirming that viable organisms are colonizing beyond the site of inoculation (vaginal vault), traveling through the cervix, and colonizing the body of the uterus. Uterine cultures were not collected in experiments B and D.

**Table 2:** *Tritrichomonas foetus* present throughout the reproductive tract of calves following intravaginal inoculation.

Calf ID	Tissue	PCR result
A.A	Vaginal vault	Positive
	Cervix	Positive
	Uterus	Positive
A.B	Vaginal vault	Positive
	Cervix	Positive
	Uterus	Positive
A.C	Vaginal vault	Positive
	Cervix	Positive
	Uterus	Positive

PCR using TfCP2 primers was performed on vaginal, cervical, and uterine tissue samples collected at necropsy from calves in experiment A.

### 3.3 Inflammation in reproductive tracts of infected heifer calves

**Figure 4:**
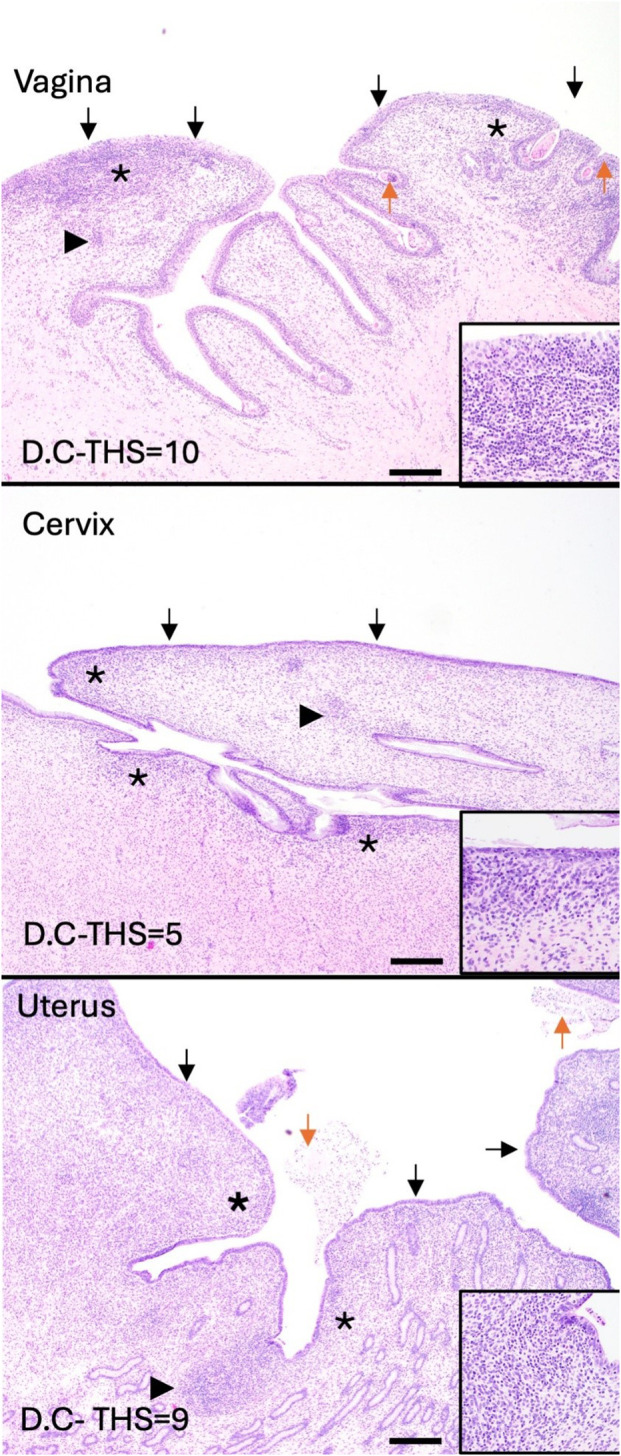
Inflammation is present throughout the reproductive tract of experimentally infected heifer calves (experiment C). All calves had at least mild inflammation in the vagina, cervix, and uterus at 42 days post inoculation. Total histologic scores (THS) were determined for each tissue sample. Representative images were selected to demonstrate the most common histologic changes in each tissue. Vaginal lesions included fibrinosuppurative intraluminal exudate (orange arrows), moderate epithelial attenuation to focal ulceration (black arrows), lymphoplasmacytic to suppurative vaginitis (asterisks), and moderate scattered submucosal lymphoplasmacytic nodules and perivascular cuffs (arrowheads). Cervical lesions included epithelial attenuation (black arrows), mild lymphoplasmacytic cervicitis (asterisks), and scattered lymphoplasmacytic nodules (arrowheads). Uterine lesions included epithelial attenuation (black arrows), mild superficial endometritis (asterisks), and increased lymphoplasmacytic to lymphohistiocytic endometrial nodules (arrowheads). Scale bars=500µm.

Tissues (vaginal vault, cervix, and uterus) from all calves in experiment D were evaluated for the following criteria: epithelial degeneration, epithelial proliferation, intraluminal exudate, inflammation severity, inflammation distribution, hemorrhage, and edema. A total histologic score (THS) was determined based on the sum of individual criteria scores for each tissue (**Table 3**). Representative lesions are shown in **Figure 4**. Moderate histopathologic changes were observed in the vaginal sections of 5/6 (83.3%) of calves. These changes were characterized by scant to small amounts of fibrinosuppurative intraluminal exudate, moderate epithelial attenuation to focal ulceration, lymphoplasmacytic to suppurative vaginitis, and moderate scattered submucosal lymphoplasmacytic nodules and perivascular cuffs. The remaining calf (D.B) had similar but less severe histopathologic lesions in the vagina. Mild histopathologic changes were present in the cervical sections of 4/6 (66.7%) calves. These changes were characterized by mild epithelial attenuation, mild lymphoplasmacytic cervicitis, and scattered lymphoplasmacytic nodules. The remaining calves had similar but more moderate inflammatory lesions. Mild histopathologic changes were present in the uterine sections of 4/6 (66.7%) calves. These changes were characterized by mild epithelial attenuation, mild superficial endometritis, and increased lymphoplasmacytic to lymphohistiocytic endometrial nodules. Of the remaining calves, one had moderate changes, and one had severe changes in the uterus that contained similar inflammatory infiltrates. All sections were negative for GMS staining, indicating the absence of fungal organisms. Two calves (D.A and D.F) had rare gram-positive cocci in luminal mucus of the uterus (D.A) and cervix (D.F). The presence and character of this bacteria was consistent with normal flora of the mucus layer.

**Table 3:** All experimentally infected calves had at mild inflammation throughout the reproductive tract.

Calf ID	Tissue	Total Histologic Score (THS)
D.A	uterus	3 - Mild
	cervix	3 - Mild
	vagina	8 - Moderate
D.B	uterus	3 - Mild
	cervix	2 - Mild
	vagina	3 - Mild
D.C	uterus	9 - Moderate
	cervix	5 - Mild
	vagina	10 - Moderate
D.D	uterus	5 - Mild
	cervix	2 - Mild
	vagina	9 - Moderate
D.E	uterus	20 - Severe
	cervix	9 - Moderate
	Vagina	9 - Moderate
D.F	uterus	4 - Mild
	cervix	10 - Moderate
	vagina	9 - Moderate

Total histologic scores were determined based on examining tissues for various criteria, both the numerical score and category (no pathologic change, mild, moderate, severe) is given for each animal included.

### 3.4 Putative *T. foetus* host cell receptor expressed by prepubescent heifer calves

**Figure 5:**
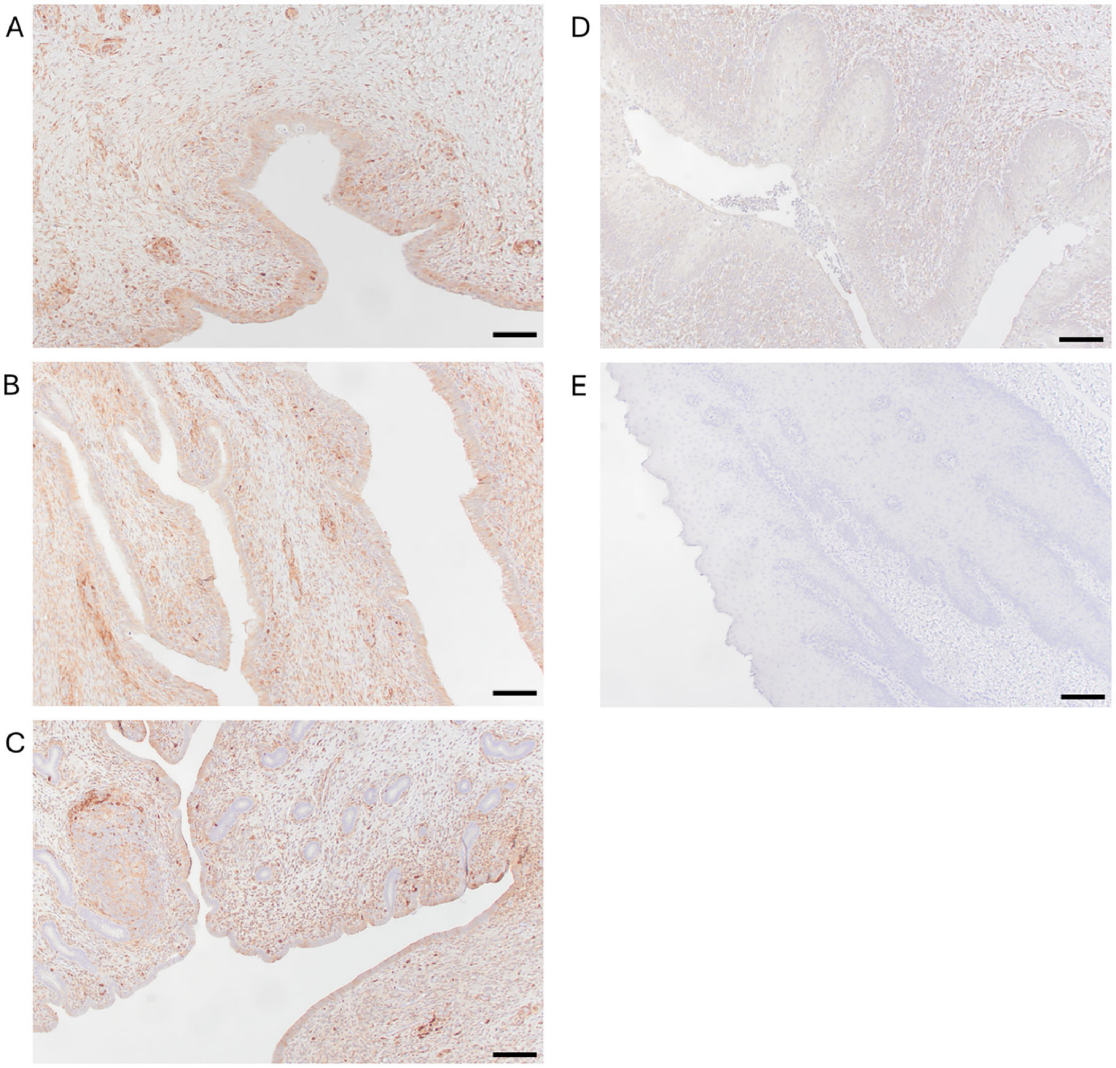
Galectin-1 is expressed throughout the reproductive tract of prepubescent calves. Immunohistochemistry using anti-galectin-1 antibody (JM13-37) with horseradish peroxidase secondary antibody demonstrates the presence of galectin-1 throughout the epithelium and submucosa of sections of vagina **(A)**, cervix **(B)**, and uterus **(C)** of calves. Peroxidase positivity is indicated by red staining. Preputial mucosa served as a positive control **(D)**. Replacement of the primary antibody (JM13-37) with PBST served as a negative control **(E)**. Scale bars=500μm. All images were captured at 100x magnification.

Immunohistochemistry staining confirmed the presence of galectin-1, a putative host cell receptor for trichomonads throughout the epithelium and submucosa of the vagina, cervix, and uterus of prepubescent heifer calves (**Figure 5**). This is the first report of galectin-1 expression in these tissues in prepubescent heifers.

## 4 Discussion

*Tritrichomonas foetus* has a significant impact on the beef cattle industry since early embryonic death results in a prolonged calving interval (Mich et al., 2016). Prolonged calving intervals decrease calf crop uniformity that is desired by cow-calf producers in order to maximize their economic outcomes (Clark et al., 1983; Rae, 1989; Collantes-Fernández et al., 2014; Ortega-Mora et al., 2022). Additionally, the cost associated with replacing a carrier bull is significant (Mich et al., 2016; Yao, 2021). While heifers and cows typically do clear the infection, the impacts of a single trichomonosis outbreak are devastating for a cattle operation.

Currently, *T. foetus* is regulated at the state level, with some states opting for strict testing and movement restrictions and other states – typically those with smaller beef cattle populations – opting to forego regulations all together (Martin et al., 2021). This results in a patchwork of regulations and the opportunity for the parasite to be spread with the movement of infected animals. Inconsistent regulations, paired with the lack of an approved treatment, allow *T. foetus* to continue to impact the beef cattle industry in the United States. Continued efforts improving diagnostic and treatment options are needed to advance control efforts for bovine trichomonosis. The logistical, financial, and safety challenges associated with housing mature cattle in research settings has severely hampered the progress toward reducing the impact of bovine trichomonosis. The present study was designed with the ultimate goal of gaining a model for the study of *T. foetus* thereby enabling studies of potential treatments, vaccines, and improved diagnostic strategies.

### 4.1 Infection of prepubertal calves was unexpectedly effective and shared characteristics with natural infection

Following infection with trophozoites directly in the vaginal vault, a majority of calves were consistently culture positive four weeks post-inoculation. Importantly, viable organisms were also present in cultures from the uterine lumen which indicated successful growth and survival. Interestingly, our results show that even in a prepubescent animal, trophozoites are able to penetrate through the cervix, whereas sperm cells are unable to do so when a cow is in a period of anestrus (Nabors and Linford, 2015). Significantly, the susceptibility of heifers in our study demonstrated that hormonally driven changes in the female reproductive tract are not a prerequisite for infection. Experimental infection studies in mature heifers and cows typically involve hormone synchronization to ensure animals are in the same stage of the estrous cycle (Stockdale et al., 2007; Fuchs et al., 2017; Ortega-Mora et al., 2022). The use of prepubescent animals removes hormonal influence and the risk of failure of synchronization attempts as experimental variables. Taken together, the series of experiments presented herein demonstrate the ability of prepubescent heifer calves to maintain *T. foetus* infections for at least 6 weeks without the need for exogenous hormones or immunosuppressive treatments. Our study reveals the exciting potential of a natural host calf model to study the biology of the parasite.

Calves were not culture positive at all time points during the study. While in experimental context, parasite culture has the benefit of confirming the presence of viable organisms, one drawback to the testing method is reduced sensitivity compared to PCR. Infection rates in this study are consistent with those reported in post-pubertal heifer infection challenge studies. Anderson et al. reported that of 6 heifers challenged with *T. foetus*, 4 were culture positive at weekly intervals, with 2 failing to produce a positive culture result (BonDurant et al., 1993; Anderson et al., 1996). Additionally, previous studies have also noted that challenged heifers may go between culture negative and positive status from week to week, meaning that a single negative culture may not reflect the true infection status (BonDurant et al., 1993). This is broadly accepted and the reasoning for the standard requirement of three, consecutive weekly negative culture samples prior to permitting the movement of bulls across state lines where *T. foetus* movement regulations are in place (Schroeder et al., 2023).

This is the first evaluation of microscopic lesions caused by *T. foetus* in prepubescent heifers. Histopathologic evaluation revealed lesions and cellular infiltrates consistent with findings in previous studies in sexually mature cattle, including the presence of lymphoid nodules and lymphoplasmacytic infiltrates (Parsonson et al., 1976; Anderson et al., 1996). The most recent description of histopathologic changes associated with *T. foetus* infections in cattle was observed nearly 30 years ago (Anderson et al., 1996). Gram and GMS staining confirmed that inflammation was not the result of fungal or bacterial pathogens and therefore supported the hypothesis that the observed lesions and inflammation were due to *T. foetus*. Observing similar microscopic lesions in calves and sexually mature cattle further supports the utility of our infection model.

The presence of galectin-1 throughout the reproductive tract of prepubescent is a novel finding. This lectin is known to be involved in adhesion of the closely related human pathogen, *Trichomonas vaginalis*, to host cells via parasite lipophosphoglycan (LPG) (Okumura et al., 2008; Ryan et al., 2011). It is known that lipophosphoglycan moieties present on *Tritrichomonas foetus* are involved in the adhesion process, but the specific host cell receptors remain elusive (Singh et al., 1999). The present study demonstrated galectin-1 expression in the reproductive tract of 6-week-old heifer calves but did not determine if it was essential for parasite adhesion. A recent study also confirmed that galectin-1 was expressed in the penis and prepuce of prepubescent bull calves (Martin et al., 2025). Further investigation is needed to determine if galectin-1 is essential for adhesion *T. foetus* infection in cattle.

There was a degree of biological variation among our 4 experiments. For example, the proportion of culture positive calves 4 weeks post-inoculation in experiment D was lower than experiments B and C, with 60% (experiment D) and 83% (experiments B and C) culture positive calves at this timepoint. It is important to note that six out of twelve calves in experiment D experienced clinical signs unrelated to *T. foetus* and consistent with *Mycoplasma* infection (fever, nasal discharge, head tilt). These animals were treated with antibiotics (tulathromycin) and NSAIDS (meloxicam) and their symptoms subsequently resolved. Three of the six calves treated for *Mycoplasma* were *T. foetus* culture negative 4 weeks post-inoculation, accounting for 75% of the culture negative calves in experiment D. Five out of six calves without *Mycoplasma* were culture positive at 4-weeks post inoculation, consistent with the results of experimental groups B and C. We suspect that pyrexia, and not antibiotic treatment, led to reduced infection rates/durations in this experimental group since there is no evidence *T. foetus* is susceptible to tulathromycin and *T. foetus* has poor antimicrobial susceptibility (Sui et al., 2024). Prophylactic antibiotic treatment for pre-existing bacterial infections, prior to parasite inoculation, may be indicated to ensure optimal infection with *T. foetus* in future studies.

### 4.2 Limitations of this study

The primary constraints to interpretation of the present report are variations in methods across and within experimental groups. Many of the variations were due to unplanned events and are inherently related to the logistics of conducting studies in cattle in a research environment. Such studies are subject to sudden changes as well as long term planning efforts due to extended gestation periods, fluctuations in cash markets, availability of facilities, and unexpected concomitant disease. These factors tend to be more difficult to control in cattle compared to the level of control that is possible for environmental control in rodent studies. Herein, we have summarized a collection of experiments carried out across a period of several years. While some variables vary slightly across individual experiments, we aimed to present all experiments together in a single cohesive report rather than deliver our results in a patchwork of publications which could be more difficult to interpret.

Experiment A served as the pilot study for this project and was planned to be short-term in order to determine the feasibility of pursuing future experiments. Experiment B aimed to determine the ability of the calf model to maintain longer term infections to demonstrate the potential of the model for use in treatment and vaccine studies. In experiment B the decision to reinfect animals following multiple negative culture results was made in an effort to increase the number of animals that were culture positive at necropsy. Consistent with natural infection, culture and PCR results can lead to intermittent positives, and this was observed in some studies following a single inoculation (**Supplementary Figure S1**). While we were aware of this phenomenon, we elected to re-inoculate selected calves in experiment B. Our intention was to maximize the number of tissues from culture positive animals which could be utilized for downstream applications such as histology. Although this was not ideal, attempting to ensure all calves were positive at necropsy was necessary to decrease the overall number of animals used in our studies. Experiments C and D were more consistent in approach with regularly spaced sampling time points. The number of animals in experiment D (n=12) necessitated two separate days for necropsy and sample processing. Ideally, the endpoint of the experiment would have been the exact same for all animals.

It should be noted that we did not use the exact same number of trophozoites across all four experiments. The underlying rationale was that we used a vast excess of trophozoites and we used the maximum number of log-phase parasites available to us at the time cohorts of calves became available. When growing trophozoites we seeded culture medium with consistent numbers of trophozoites from the same passage of liquid nitrogen seed stocks, variation in cell revival and replication led to differences in the number of organisms available on planned inoculation days. Due to facility availability and scheduling, we elected to move forward with inoculation rather than attempt to grow additional trophozoites. We did, however, ensure that parasites were in log-phase of growth with vigorous motility prior to infection. The minimum number of *T. foetus* trophozoites under natural conditions is not known. However, insights regarding infectious dose could be further resolved using our model since it would be unlikely to determine the total number of organisms inhabiting the prepuce of a bull under natural conditions. In our case, for ethical reasons, we elected not to sacrifice additional animals for this purpose alone.

Housing for the calves in this study varied among the four experiments. Experimental groups A and B were housed indoors while groups C and D were house outdoors. Housing decisions were based in part on facility availability as well as a preference for outdoor housing after pilot studies demonstrated the tractability of this model under more controlled conditions. *Tritrichomonas foetus* is not known to survive outside the reproductive tract, so environmental contamination is highly unlikely to have influenced our results. Likewise, the cervix of prepubertual heifers is closed, providing a relatively sterile uterine environment. Histological examination of tissues did not reveal additional microbial infection that could have been introduced from the environment. Studies aiming to study the impact of environmental conditions, co-infections, and the vaginal microbiome could be made possible using our model.

### 4.3 Applications and future directions

In addition to the benefits of this model for advancing the study of *T. foetus* in the bovine host, this model may also be beneficial in the study of *Trichomonas vaginalis*, the cause of human trichomoniasis. Trichomoniasis is the most common non-viral sexually-transmitted infection globally (Van Der Pol, 2007). Currently, human trichomoniasis research relies on a *T. foetus* infected mouse model (Nieskens et al., 2024). The calf infection model would allow for inferences to be made in a natural host, rather than a rodent model. Natural host models can provide a more accurate representation of the disease and more physiologically relevant information than rodent models. Cattle are known to be good models for human reproductive system research including research regarding placental insufficiency and ovarian function (Pramod et al., 2025). In female *T. vaginalis* infections, inflammation of the reproductive tract is characterized by the presence of lymphocytes and macrophages, similar to the findings in this study and other *T. foetus* infection studies (Edwards et al., 2016). Histologically, the human and bovine uterus share the presence of simple columnar epithelium with ciliated and secretory cells dispersed throughout the epithelium (Gálfiová et al., 2022; Senn et al., 2024). The dominance of *Lactobacilli* spp. in the female reproductive tract is unique to humans and would present one disparate factor in the bovine model (Miller et al., 2016; Gupta et al., 2024). Comparative pathology studies are needed to evaluate the utility of a *T. foetus* calf model as a surrogate for human trichomoniasis and confirm advantages over the currently used mouse infection model.

Our studies also highlighted the need to optimize a method for quantitating severity of *T. foetus* infection. While we made efforts to collect samples in a similar manner, parasite culture in TYM led to trophozoite counts that varied significantly among positive animals (e.g., 1 vs 1,000,000 trophozoites/mL). Based on the methods employed in this study, it is unclear if the observed level of variation reflects biological differences among infected calves or is simply related to the limits of our sampling methods. Swab depth, duration, and number of rotations could all impact the quality of the sample and should be evaluated. Clinically, status of infection is simply characterized as positive or negative, and the importance of trophozoite numbers on disease severity or progression is not established. However, quantification of infection is important for assessing success of parasitological studies or interventions such as treatment and vaccination. Possible methods to achieve this could include cycle threshold (CT) values for qPCR, lesion scoring, or quantification of *T. foetus* antigen via ELISA. While some of these methods have been used to detect parasites from naturally infected animals, their use in quantification of disease is not well-established. Future studies in our model will aim to investigate infectious dose as well as quantification of parasite burden with lesion severity.

Our findings will also enable us to address new questions regarding characterization and optimization of a heifer calf model for bovine trichomonosis. Future studies should aim to evaluate the duration of infection, as the results of these studies did not extend beyond 6 weeks. With 83% of calves remaining culture positive at 6 weeks, it is unclear if heifer calves can maintain infections far beyond this timepoint. While the paradigm is that cows are only infected transiently, there are some reports of persistently infected cows (BonDurant, 2005). The present study utilized a single, high-dose inoculum. The optimal inoculation regimen and dose should be explored to aid in the optimization of this model. Our experiments relied on a single field strain (IA-1), of *T. foetus*. Ideally, infection challenges with other parasite strains should be performed and whereby infection dynamics of various strains could be compared. Lastly, these experiments utilized cross-bred dairy calves. It is possible that differences in breed susceptibility exist, and thus experimental infection of additional cattle breeds could be explored to aid in model optimization. While some optimization is needed, the success of our experiments underscores a new ability to answer research questions that were previously difficult to obtain.

In this study, we tested the hypothesis that heifer calves are a suitable natural host model for bovine trichomonosis and challenged the dogma that estrus and ovulation are required for infection of the bovine female reproductive tract with *T.foetus*. The results of our series of experiments supported our hypothesis and demonstrate significant promise of the heifer calf model. The ability of calves to reliably maintain infections for at least 6 weeks with viable organisms colonizing and replicating in the reproductive tract is a significant finding. Our natural host model fills a significant knowledge gap and allows for further understanding of the parasite-host relationship, as well as providing a straightforward method to assess novel treatments, vaccines, and diagnostic platforms.

## Data Availability

The original contributions presented in the study are included in the article/**Supplementary Material**. Further inquiries can be directed to the corresponding author.
