# Supplementary material for: Infection of prepubertal heifer calves as a natural host model for Tritrichomonas foetus
Source: Front Cell Infect Microbiol. 2025 Oct 15;15:1628192. doi: 10.3389/fcimb.2025.1628192 (PMC12568593; doi:10.3389/fcimb.2025.1628192)
Supplement: Supplementary file 1 [file DataSheet1.docx]

**Supplemental Figure 1.** Complete sampling schedule and corresponding culture results for all experimental groups. Calves in all experimental groups were sampled by swabbing the vaginal vault with a TYM-soaked cotton tipped applicator. Gray cells indicate calves that had been euthanized prior to the indicated timepoint. A separate subfigure is provided for each individual calf experiment (A-D).
